# The Role of *BRCA1/2*-Mutated Tumor Microenvironment in Breast Cancer

**DOI:** 10.3390/cancers13030575

**Published:** 2021-02-02

**Authors:** Svetlana Miklikova, Lenka Trnkova, Jana Plava, Martin Bohac, Marcela Kuniakova, Marina Cihova

**Affiliations:** 1 Cancer Research Institute, Biomedical Research Center, University Science Park for Biomedicine, Slovak Academy of Sciences, 84505 Bratislava, Slovakia; svetlana.miklikova@savba.sk (S.M.); lenka.trnkova@savba.sk (L.T.); jana.plava@savba.sk (J.P.); 2 2nd Department of Oncology, Faculty of Medicine, Comenius University, National Cancer Institute, Klenova 1, 83310 Bratislava, Slovakia; bohac.md@gmail.com; 3 Department of Oncosurgery, National Cancer Institute, Klenova 1, 83310 Bratislava, Slovakia; 4 Regenmed Ltd., Medena 29, 81108 Bratislava, Slovakia; 5 Institute of Medical Biology, Genetics and Clinical Genetics, Faculty of Medicine, Comenius University, Sasinkova 4, 81108 Bratislava, Slovakia; marcela.kuniakova@fmed.uniba.sk

**Keywords:** breast cancer, tumor microenvironment, *BRCA1/2* mutations, stromal cells, epithelial-to-mesenchymal transition, angiogenesis

## Abstract

**Simple Summary:**

The link between *BRCA1/2* mutations and high susceptibility to breast cancer development has been well-established for years. However, the potential impact of *BRCA1/2* mutations on the individual cell populations within the unique tumor microenvironment and their relation to breast cancer has been understudied. This review aims to provide significant insights into up-to-date knowledge about the role of *BRCA1/2*-mutated tumor microenvironment and possible mechanisms by which it interacts with and promotes breast cancer development and progression. Uncovering and exposing these mechanisms of the aberrant microenvironment might provide more effective strategies for treatment of germline mutation-carrying breast cancer patients.

**Abstract:**

Taking into account the factors of high incidence rate, prevalence and mortality, breast cancer represents a crucial social and economic burden. Most cases of breast cancer develop as a consequence of somatic mutations accumulating in mammary epithelial cells throughout lifetime and approximately 5–10% can be ascribed to monogenic predispositions. Even though the role of genetic predispositions in breast cancer is well described in the context of genetics, very little is known about the role of the microenvironment carrying the same aberrant cells impaired by the germline mutation in the breast cancer development and progression. Based on the clinical observations, carcinomas carrying mutations in hereditary tumor-suppressor genes involved in maintaining genome integrity such as *BRCA1/2* have worse prognosis and aggressive behavior. One of the mechanisms clarifying the aggressive nature of *BRCA*-associated tumors implies alterations within the surrounding adipose tissue itself. The objective of this review is to look at the role of *BRCA1/2* mutations in the context of breast tumor microenvironment and plausible mechanisms by which it contributes to the aggressive behavior of the tumor cells.

## 1. Introduction

Breast cancer represents a global burden and is considered a leading cancer type and the most common reason of cancer related deaths among the women worldwide. In 2020, it was estimated that breast cancer accounts for 30% of newly diagnosed cancer cases and 15% of cancer-related deaths in women [1]. Breast carcinoma includes several types that differ in the site at which they appear and their invasive potential. While breast carcinoma in situ refers to epithelial neoplasia that is limited to the breast duct or lobule and is considered as non-invasive or pre-invasive stage, invasive breast carcinoma describes the state when cancer cells have penetrated through the basement membrane and spread from ductolobular system to adjacent stroma. The main histological variants among the invasive breast cancers include invasive ductal and invasive lobular carcinoma with invasive ductal carcinoma being the most common type accounting for approximately 80% of invasive breast cancers [2,3]. Regarding in situ breast cancer, ductal carcinoma in situ represents the majority of diagnosed in situ breast cancers and it is considered as a precursor for development of invasive breast cancer [4]. Lobular breast carcinoma in situ is being discussed for its potential as a precursor, however, it rather indicates an increased risk for development of invasive breast cancer [5]. According to the current molecular classification, breast cancer is divided into several molecular subclasses based on gene expression profiles. The luminal A and luminal B subtypes are characterized by a gene expression pattern of luminal epithelial cells, with the luminal A subtype referring to tumors positive for estrogen receptor (ER) and/or progesterone receptor (PR) expression, negative for human epidermal growth factor receptor 2 (HER2) expression and with low levels of a proliferation marker Ki67. The luminal B subtype may show more increased expression of HER2 than the luminal A type and high Ki67 levels. HER2-enriched tumors show high HER2 expression. Basal-like breast cancer is considered as more aggressive type and is characterized by gene expression of basal epithelial genes, with tumors negative for ER and PR expression and with low HER2 expression [6]. ER-, PR- and HER2-negative tumors are referred to as triple negative breast cancer (TNBC), although these tumors include both basal-like and non-basal tumors [7]. Depending on the genetic background, the majority of breast cancer cases are sporadic with no related family cancer incidence. Sporadic breast cancer usually develops in later stages of life as a result of multiple acquired somatic mutations. However, approximately 15% of breast cancers are classified as familial with a patient´s first or second degree relative being affected [8]. Except for the genetic predisposition, familial cancers may be influenced also by non-genetic and environmental factors shared among the relatives [9]. Five to 10% of the breast cancer cases are linked with heritable germline mutations in the breast cancer related genes [10,11]. Currently, more than 25 genes have been associated with hereditary breast cancer and nearly all of them are tumor-suppressors that participate in genome stability pathways, particularly in homologous recombination DNA repair pathway and to some extent also mismatch repair, as well as inter-strand DNA crosslink repair [12]. These genes include, among others, well-described and highly penetrant tumor suppressors *BRCA1* and *BRCA2*. Mutations in these genes were described in approximately 25% of hereditary breast cancers [13]. Estimated cumulative risk for development of breast cancer in carriers of *BRCA1* and *BRCA2* mutation is 72% and 69%, respectively, up to the age of 80 years [14]. *BRCA1* mutation mostly accounts for the development of TNBC with basal-like phenotype and high proliferation rate [15,16,17,18]. On the contrary, carriers of *BRCA2* mutation are more prone to develop ER- or PR-positive breast cancer [16]. The function of *BRCA1/2* is mostly ascribed to the maintenance of genome stability through participation in DNA repair processes including homologous recombination (a key error-free DNA repair mechanism involved in double strand break repair), stabilization of DNA replication fork [19], regulation of transcription [20] and participation in DNA damage checkpoints [21] (reviewed in Nielsen et al., 2016 [12]).

BRCA1 and BRCA2 form various complexes with different proteins helping to safeguard the genome via damage signal mediation and initiation of repair by the effectors (reviewed in Savage et. al., 2015 [22], Roy et. al., 2011 [23], and summarized in Table 1).

Besides the *BRCA1* and *BRCA2,* mutations in other rare and highly penetrant genes are linked with increased risk of breast cancer development. These include *STK11* (serine/threonine kinase 11; mutation is also referred to as Peutz–Jeghers syndrome) [33], *PTEN* (phosphatase and tensin homolog; mutation is referred to as Hamartoma-tumor syndrome) [34], *TP53* (Li–Fraumeni syndrome) [35], or *E-cadherin 1* (*CHD1*; mutation predisposes its carrier to hereditary diffuse gastric cancer with increased risk of lobular breast carcinoma) [36] and genes with moderate penetrance such as *CHEK2*, *BRIP-1*, *PALB2* or *ATM* (reviewed in Shiovitz and Korde, 2015 [37]). Low-risk variants account for 18% of familial relative risk as identified through genome wide association study [38].

The link between *BRCA1/2* mutations and high susceptibility to breast cancer development has been well-established for years. However, the potential impact of *BRCA1/2* mutations on the individual cell populations within the unique tumor microenvironment (TME) and their relation to breast cancer has been understudied. The specific role of TME in all aspects of breast cancer “life cycle”, from initiation, through progression to gaining highly aggressive and metastatic phenotype is now undoubtedly one of its key hallmarks. If there is a difference between TME of sporadic and hereditary breast cancer, it has not been comprehensively addressed. Nevertheless, understanding the role of these mutations in the context of unique TME can help to better stratify the patients in terms of tumor development risk and can lead to establishment of new potential therapeutic targets by which we would be able to affect the tumor aggressiveness. The objective of this review is to look at the role of *BRCA1/2* mutations in the context of TME and plausible mechanisms by which the mutation-carrying TME might contribute to aggressive behavior of the tumor cells.

## 2. Breast Tumor Microenvironment: Modulator of Tumor Initiation, Progression, Metastasis and Therapy Response

The exact mechanisms responsible for cancer initiation, progression and metastasis are still not clear. The TME, composed of non-cancer cells, has been recognized as a major factor influencing the regulation of cancer cell growth, determining metastatic potential and impacting the outcome of therapy. While the stromal cells are not malignant per se, their role in influencing the tumor biology is so crucial to the survival of the tumor that these non-cancer tumor-surrounding cells have become an attractive target for therapeutic agents. Moreover, TME is now considered to be a hallmark of cancer biology [39,40,41].

The heterogeneity of TME depends on the location within the tumor. Cells forming tumor stroma, a critical component of the TME, may significantly differ at the tumor periphery and within the tumor core [42]. This is partially due to the randomly generated mutations within the tumor cells, immune cells infiltration, tumor cell necrosis and interstitial pressure [43]. Naturally, each tumor has its own unique TME, which is comprised also of non-cellular components such as extracellular matrix (ECM), soluble factors (e.g., cytokines, hormones, growth factors and enzymes) and physical properties (e.g., pH and oxygen content), which also affect properties of the TME [41]. Various cell types are involved in the bidirectional tumor-stroma interactions, mostly cancer-associated fibroblasts (CAFs), endothelial cells and pericytes, immune cells, surrounding adipocytes and mesenchymal stromal cells (MSCs), which may be resident or attracted by the tumor from distant sites [44].

CAFs are one of the most important key players in the TME. They can affect various cancer cells characteristics, such as proliferation, invasion, angiogenesis, inflammation, immunosuppression or chemoresistance. There is a remarkable heterogeneity between CAFs populations due to the expression of different surface markers indicating the different origin of the cells. They can originate from tissue resident fibroblasts, myofibroblasts, MSCs, adipocytes, pericytes, vascular smooth muscle cells, epithelial and endothelial cells (reviewed in Ting Lee et al., 2020 [45]). MSCs, which can differentiate into the CAFs in the TME, are another key part of the breast tumor stroma, as adipose tissue is one of their main sources (reviewed in Ullah et al., 2015 [46]).

Many experimental studies confirmed that MSCs have the capacity to interact with breast cancer cells (reviewed in Kucerova et al., 2011 [47]) and regulate the TME. They were shown to promote breast cancer progression and metastatic spread [48,49] as well as epithelial-to-mesenchymal transition (EMT) [50,51]. On the other hand, the reports focusing on the influence of MSCs on chemotherapy response are still somehow controversial, but they show that MSCs are also able to affect the chemoresistance of breast cancer cells [52,53,54,55,56,57]. Rapid cellular proliferation and high oxygen consumption rate results in elevated nutrient demand by the carcinoma. Tumor-associated angiogenesis, a process in which endothelial cells and pericytes build new blood vessels, occurs rapidly after the tumor formation [58]. Conversion from normal endothelial cells to the tumor-associated cells is driven by many signaling pathways modified by aberrant expression profiles. Cancer-associated endothelial cells were found to express plenty of different molecules associated with increased cancer cell survival and chemoresistance [59]. Thus, these cells that line the tumor blood vessels are important targets in cancer therapies [60].

The tumor-induced systemic changes in immune cells were also associated with cancer progression and metastasis [61]. As the metastatic process is highly inefficient, circulating tumor cells (CTC) interact for example with neutrophils by forming CTC–neutrophil clusters via vascular cell adhesion molecule 1 (VCAM-1) to expand metastatic potential [62]. It was shown that association of CTC with the complete blood count (CBC)-derived inflammation-based score (monocyte-to-lymphocyte ratio or neutrophil-to-lymphocyte ratio) may help to predict overall survival and improve the prognostication of breast cancer patients [63,64].

For further insights in tumor development and therapeutic approaches, it is important to gather more information and better understand the interplay between specific components of the TME, the associated cellular communication processes and the resulting functions of this network between cancer cells and the various tumor-associated cell populations. This might be important and shed new light in understanding germline mutation-carrying tumors, where the germline mutation affects all cells in the TME. Up to now, not much attention has been paid to how the cellular components of the TME carrying the germline mutation, such as the *BRCA1/2* gene, might affect the development of breast cancer and its progression.

## 3. *BRCA1/2*-Deficient Tumor Microenvironment

The necessity to look “outside the box” of epithelial cells and search in the surrounding TME in order to elucidate *BRCA1* functions and to answer questions regarding its link to more aggressive breast tumors (high expression of nuclear grade, large tumor burden, more aggressive progression and worse prognosis) has been tackled more than 13 years ago [65]. In breast cancer, it seems that heterozygous *BRCA1*-mutated microenvironment in germline *BRCA1* mutation carriers may significantly contribute to breast cancer development by creating a pro-tumorigenic niche [66]. Several studies indicate that loss of BRCA1 in breast epithelial cells may substantially affect stromal cells residing in the TME which in turn can enhance the metastatic potential of *BRCA1*-deficient tumor cells [65,67,68]. This was confirmed by Plava et al. who compared in immunodeficient mouse model the co-injection of breast tumor cell line with MSCs obtained from different mammary gland sites of *BRCA1* germline mutation-carrying patient [48]. MSCs from breast adipose tissue where relapsed invasive ductal carcinoma was confirmed compared to MSCs from breast adipose tissue of contralateral breast where prophylactic mastectomy was performed, increased the aggressive phenotype of the co-injected tumor cells and augmented tumor volume.

Other study showed that *BRCA1*-deficient breast cancer cells can transform CAFs to their altered activated phenotype, which the authors named metastasis associated fibroblasts (MAFs). MAFs can subsequently induce metastatic changes in the breast cancer cells and accompany them during metastasis [69]. It was previously shown that BRCA1 colocalizes with Ezrin, Moesin, Radixin and F-actin and controls cell motility [70]. In connection with this information, MAFs induce changes in proliferation, invasion and migration of cancer cells, which are linked to the elevated expression of EMT markers, Ezrin and CCL5 in these cells [69].

Besides the *BRCA* mutations per se, proteins encoded by alternative *BRCA1* mRNA transcript can alter tumor microenvironment. BRCA1-IRIS, also known as IRIS (In-frame Reading of BRCA1 Intron 11 Splice variant), is an oncogene produced by the alternative splicing of the *BRCA1* mRNA. Its overexpression stimulates DNA replication [71]. Therefore, IRIS expression is high in all breast cancer subtypes and even higher in TNBC. IRIS-overexpressing TNBC cells secrete interleukin 6 (IL-6) which activates STAT3, AKT, and ERK/MAPK signaling in MSCs. Thus, IRIS-overexpressing TNBC cells are able to recruit MSCs and activate them through enhancing their proliferation and migration [72].

**Figure 1:**
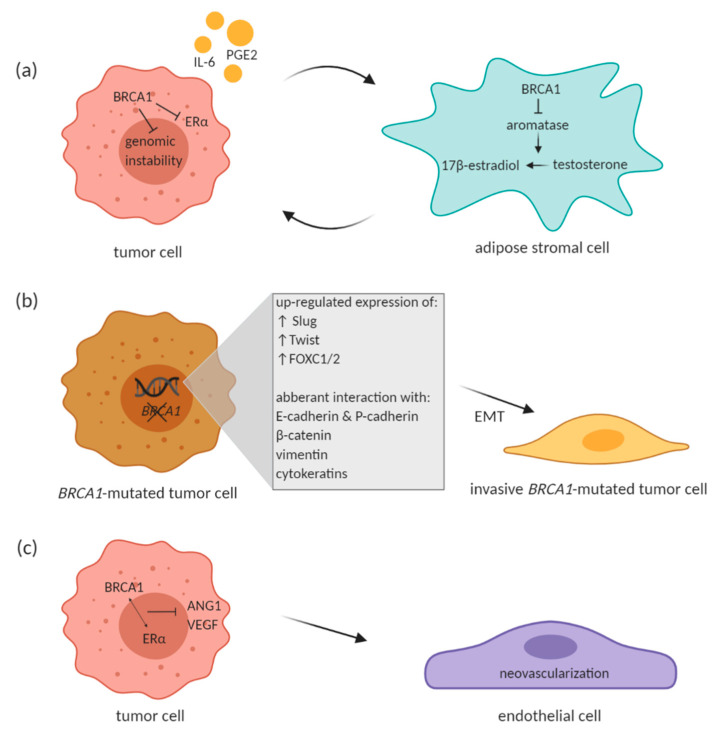
Proposed effect of BRCA1 on different cells in the tumor microenvironment (TME). (**a**) Paracrine loop between BRCA1-proficient breast tumor cell and adipose stromal cell. (**b**) *BRCA1* mutation leads to enhanced EMT phenotype. (**c**) Effect of BRCA1 on endothelial cells within the TME. BRCA1 in interaction with estrogen receptor α (ERα) has been shown to inhibit VEGF and angiopoetin-1 (ANG1) transcription. Created with BioRender.com.

McCullough et al. have pointed out to an intricate paracrine loop that exists between tumor and surrounding adipose stromal cells, which facilitates breast cancer progression in the mammary tissue microenvironment. Tumor cells produce factors such as IL-6 and prostaglandin E2 (PGE2) that stimulate aromatase expression in adipose stromal cells. Aromatase catalyzes estrogen production in the stromal cells, which in turn promotes estrogen-dependent growth of tumor cells. BRCA1 comes into the picture of this paracrine loop with its role in repressing aromatase gene expression in the stromal cells [65]. BRCA1 expressed in stromal cells may lead to reduced estrogen-mediated gene expression along with BRCA1-mediated repression of estrogen receptor alpha (ERα) in mammary cells and thus suppress the estrogen-dependent tumorigenesis (Figure 1a). If there is impaired *BRCA1* expression in the stromal cells due to the inherited mutation, the local estrogen levels are elevated and may contribute to potential tumorigenesis by increasing genetic instability. This might seem a bit striking since *BRCA1*-associated tumors are largely ERα-negative. Wang et al. suggested that estrogen promotes initiation and progression of estrogen receptor negative *BRCA1*-deficient tumors through stimulation of cell proliferation and activation of EMT. This is dependent on protein kinase B (AKT) activation and independent of ER. Authors showed that estrogen activates the AKT pathway in *BRCA1*-deficient mammary tumors by enhancing the expression of p-Akt, p-mTOR, p-Gsk3 β, and p-4Ebp1, downstream targets of Akt. Along with activation of the Akt pathway, estrogen promoted EMT and proliferation in *BRCA1*-deficient mammary tumor cells. *BRCA1*-deficient tumor cells treated with Akt inhibitor AZD5363 notably inhibited estrogen-enhanced expression of p-4Ebp1, p-mTor, and p-Gsk3 β, as well as Vim (EMT marker) and p-Fra1 (EMT-inducing transcription factor) expression, suggesting that AZD5363 efficiently suppresses estrogen-enhanced Akt pathway and EMT program in *BRCA1*-deficient tumor cells. Interestingly, estrogen activated EMT in *BRCA1*-deficient, but not in *BRCA1*-proficient tumor cells, independent of ER [73]. To sum up, the phosphatidylinositol 3-kinase/protein kinase B (PI3K/AKT) pathway is stimulated by estrogen in the ER-negative *BRCA1*-deficient breast cancer cells and leads to enhanced tumor growth [74].

In ER positive breast cancer cells, the impact of estrogen on EMT and the ability of ERα signaling to crosstalk with EMT regulators such as Snail and Slug has been suggested and described few years ago [75]. In a study by Bouris et al., the role of estrogen/ERα signaling in EMT in breast cancer cells was documented in an experiment, in which the ERα in MCF-7 cells was knocked-down through specific shRNA lentiviral particles. The cells changed phenotypically along with significant change in gene and protein expression of several markers typical for EMT, such as complete loss of E-cadherin, significantly induced expression of vimentin and fibronectin (mesenchymal protein markers) as well as of ΕΜΤ-related transcriptional regulators, such as ΖΕB1/δEF1 and SNAIL2/SLUG. ERα suppression also lead to altered expression of EGFR and HER2 receptor tyrosine kinases, and various extracellular matrix metalloproteinases and components of the plasminogen activation system. Compared to wild type MCF-7, the ERα-silenced MCF-7 cells exhibited enhanced proliferation, migration and invasion [76].

### 3.1. EMT Process in BRCA-Deficient Tumors

The EMT is a complex cellular process involved in embryogenesis, tissue reparation and wound healing, and also in tumorigenesis. During EMT, epithelial cells acquire mesenchymal phenotype and migratory and invasive properties [77,78,79]. There are plethora of biological events and signaling pathways involved. Regarding the carcinogenesis, changes in EMT regulatory pathways lead to more aggressive cellular phenotype—changes in the polarity of the cell, loss of cellular adhesions, detachment, migration, intravasation and ability to survive in the vascular system, extravasation, and metastasis. Therefore, EMT events are linked with progression from pre-invasive to the invasive state of cancer and metastatic disease [79]. To initiate all of that, many biological processes and molecules are involved, including the TGF-β, Notch and Wnt pathways, effects of the TME such as hypoxia and expression of different microRNAs [80]. Several transcription factors, including Snail 1/2 (also referred to as Snail and Slug), ZEB 1/2, Ets-1, FOXC1/2 and Twist affect expression of many genes that were identified as initiators of EMT [81,82].

In breast cancer, EMT has the greatest impact on basal-like tumors. Many studies showed that basal-like breast tumors represent the most aggressive and deadly breast cancer subtype with high metastatic ability [77,83]. As mentioned earlier, germline mutations in the tumor suppressor gene *BRCA1* increase the risk of developing basal-like breast tumors with high metastasis and poor prognosis [15,16,17,18,84,85]. It was shown that aberrant luminal stem cells can more likely give origin to basal-like tumors than basal progenitors [86,87,88]. Bai et al. showed that a germline mutation or mammary epithelia-specific deletion of *BRCA1* is responsible for the activation of EMT transcription factors and thus induction of EMT, dedifferentiation of luminal stem cells and expansion of basal and cancer stem cells. These events are important in development of basal-like tumors [82].

Many other studies reported the relationship between *BRCA1* mutation and EMT transcription factors. In *BRCA1*-mutated tumors, predominantly basal-like tumors, Slug was reported to be up-regulated although BRCA1 is not a transcriptional repressor [89,90]. Also Twist and FOXC1/2 are over-expressed in those types of tumors with *BRCA1* mutation, because BRCA1 acts like a direct repressor of these transcriptional factors under normal conditions [82,91]. In addition to the interaction with transcription factors, presence of *BRCA1* mutation was correlated with regulation of EMT through cell surface proteins E- and P-cadherin, cytoskeletal protein such as β -catenin, vimentin and cytokeratins (Figure 1b) (reviewed in Sengodan et al., 2018 [81]). As mentioned above, BRCA1 was also described to reduce breast cancer cell migration through ubiquitination of ezrin–radixin–moexin protein complex that is important in regulation of cellular motility and spreading [70].

The role of EMT in *BRCA2*-deficient tumors was correlated with primary resistance or relapse after PARP inhibitor treatment associated with rapid expression of EMT-associated markers in a treatment-dependent manner [92]. EMT-like phenotype correlated with a high expression of the *ABCB1B* gene and was associated with multidrug resistance also in *BRCA2*-deficient sarcomatoid mammary tumors. *ABCB1B* gene encodes for drug efflux transporter P-glycoprotein and its inhibition partially re-sensitized sarcomatoid tumors to the PARP inhibitors [93].

### 3.2. Impact of BRCA Deficiency on Tumor Neovascularization

Studies have shown that intricate interaction between *BRCA1*-deficient tumor cells and the surrounding stroma may also be manifested in promoting endothelial cell survival and vascularization. Tumor growth and progression is accompanied by development of vascular network within the tumor. Formation of new vasculature from existing vascular network, a process called angiogenesis, is one of the hallmarks of growing tumors and its onset is also known as angiogenic switch. Angiogenesis is under the control of number of pro- and anti-angiogenic factors like angiogenic vascular endothelial growth factor (VEGF), angiopoietins (Ang) or basic fibroblast growth factor (bFGF). Anti-angiogenic factors include for example thrombospondins or circulating endostatin and angiostatin (reviewed in Huang and Bao, 2004 [94]).

Extensive cancer cell proliferation demands higher oxygen utilization and together with inadequate blood supply within the tumor results in formation of hypoxia which is often observed in many tumors. Hypoxic conditions drive increase in activity of hypoxia inducible factors (HIF) [95]. HIF1α (a subunit of HIF1 heterodimer) is under normoxic conditions ubiquitinated and degraded by proteasome, although its expression is in cancer cells elevated during hypoxia [96]. Accumulated HIF1α dimerizes with HIF1β subunit and, together with its co-activator proteins, HIF1 participates in activation of its target genes [95]. Among them, VEGF has been widely described as a key component of angiogenic regulation and blood vessel formation [97]. VEGF, together with HIF1α, are also considered as important factors involved in cancer progression and dissemination [98,99].

Increase in VEGF and HIF1α expression has been observed in *BRCA1/2*-related and hereditary breast cancer when compared to sporadic breast cancer as well as in *BRCA1/2*-related hereditary breast cancer compared to other types of hereditary breast cancer [100]. Moreover, BRCA1 in interaction with ERα has been shown to inhibit *VEGF* transcription and protein expression through estrogen signaling pathway (Figure 1c). BRCA1 in interaction with ERα prevented VEGF expression by its binding to VEGF promoter and thus suppressed its activity [101]. Elevated expression of Ang-1, Ang-2 and VEGF was also described in *BRCA1/2*-mutated tumors of hereditary breast cancer patients [102]. Similarly, mammary tumors from *BRCA1*-deficient mice exhibited increased expression of Ang-1 together with notable vascular growth [103]. In this study, BRCA1 was also described to inhibit Ang-1 transcription by forming a repressive complex with CtIP (CtBP-interacting protein) and ZBRK1 (Zinc finger and BRCA1-interacting protein with KRAB domain 1). This complex then represses Ang-1 through ZBRK1 recognition site on Ang-1 promoter [103]. Moreover, Danza et al. highlighted involvement of miRNA-mediated angiogenesis in *BRCA1/2*-deficient tumors. The authors showed miR-578 and miR-573 involvement in *BRCA1/2*-related angiogenesis by affecting VEGF, FAK and HIF-1 signaling pathways [104]. The above-mentioned studies therefore suggest an involvement of BRCA1/2 in tumor angiogenesis and cancer progression.

## 4. Other Observations from an Altered Tumor Microenvironment

One of the mechanisms clarifying the aggressive nature of *BRCA*-deficient tumors implies alterations within the surrounding adipose tissue itself. As it has been described earlier, adipose-derived stem cells from the tumor-surrounding adipose tissue contribute to tumor cell proliferation and invasion. One of the studies proposed that *BRCA1* mutation-carrying adipose-derived stromal cells have defective DNA repair ability, therefore accumulate DNA damage, which leads to active ATM complex. As a con-sequence, p21 is activated and *BRCA1*-deficient adipose-derived cells acquire senescent phenotype and secrete increased number of inflammatory cytokines, which promote breast tumor proliferation and invasion [105].

Another study showed that *BRCA1*-deficient fibroblasts displayed increased cell proliferation, markers of both autophagy and mitophagy, and in chemical pseudo-hypoxic conditions they expressed elevated levels of HIF-1α. Due to the elevated production of ketone bodies they can increase mitochondrial activity in cancer cells. Importantly, *BRCA1*-deficient fibroblasts induced more than 2-fold increase in tumor growth compared to the tumors where control fibroblasts were injected with cancer cells. Therefore, the authors conclude that BRCA1 deficiency in tumor stroma metabolically promotes cancer progression via ketone production [106].

It has also been outlined by functional in vitro experiments that *BRCA1*-heterozygous cells create a proliferation-favoring environment compared to wild type adipose stromal cells, thus leading to tumor development enhancement [66]. But further insights need to be gathered to elucidate exact mechanisms. Noteworthy observations were obtained by performing whole-genome analysis of *BRCA1/2*-related invasive breast carcinomas. This analysis revealed that loss of heterozygosity (LOH) or allelic imbalance (AI) was in the cancer stroma very similar to that in epithelium in the hereditary breast cancer patients. This was remarkably different from sporadic breast tumors, where the LOH/AI in the epithelium far exceeded these events in the tumor stroma. In a subset of samples, enriched for *BRCA1* cases, overall LOH/AI was even higher in the stroma than in the epithelium. These observations strongly indicate that accumulation of genomic instability through LOH or AI in the tumor stroma coincides with that in the cancer epithelium and might facilitate a microenvironment which subsequently might lead to neoplastic transformation [68]. Thus, mutations in adjacent stromal cells might create an aberrant and permissive microenvironment allowing outgrowth of premalignant cells. The importance of tumor stroma in hereditary breast carcinogenesis may be indirectly indicated in an observation that germline mutation-carrying women who undergo prophylactic mastectomy without previous carcinoma history have an altered breast lobular architecture. Compared with the controls, who have a denser and fibrotic intralobular stroma, these patients have less differentiated lobules [107].

It is difficult to forecast to what extent germline variants affect tumor progression and clinical outcome. However, Milanese et al. developed a model to predict clinical outcome of breast cancer patients using germline variants with specific gene signatures predominately enriched in T cell function, antigen presentation, and cytokine interactions that can favor pro-tumorigenic environment via alteration of immune system functions and the immune tumor microenvironment. These gene signatures derived from the genes containing functionally germline variants were shown to distinguish recurred and non-recurred patients in two ER+ breast cancer independent cohorts [108].

## 5. Future Prospects and Therapeutic Strategies for *BRCA*-Associated Breast Cancer

**Figure 2:**
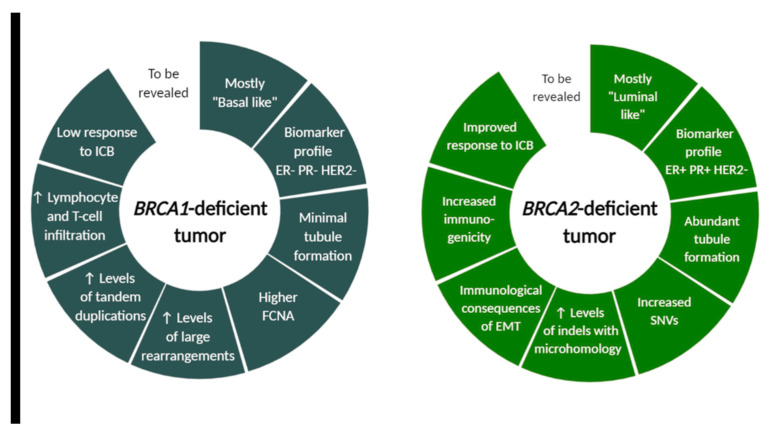
*BRCA1*- and *BRCA2*-deficient tumor characteristics differ in morphological, molecular and immunological features. Although many tumor cell-intrinsic differences between *BRCA1* and *BRCA2*-associated cancer were revealed, this field still warrants further investigation for successful design of future clinical trials. FCNA (fraction copy-number alterations), ICB (immune checkpoint blockade), SNVs (single-nucleotide variants), indels (insertions and deletions), EMT (epithelial-to-mesenchymal transition). Created with BioRender.com.

Carriers of mutations in tumor-suppressor genes carry a lifelong risk of developing cancer. The most effective prevention strategy and cancer risk management option for *BRCA* carriers is preventive surgery. A risk reduction surgery such as prophylactic mastectomy can offer areolar-sparing or nipple sparing mastectomy with immediate reconstruction by utilizing implants or autologous abdominal tissue. Other prevention strategies involve chemoprevention with selective estrogen receptor modulators such as tamoxifen or raloxifene or an aromatase inhibitor such as exemestane, and breast cancer screening with mammography and/or MRI [109,110]. Development of more effective therapeutic management, however, requires complex understanding of the key mechanisms in hereditary breast cancer. Recent studies suggest that cancer predisposition, especially in hereditary breast cancer syndrome, cannot be solely explained by the defects in DNA repair, but other factors are most likely at play. The significant role of TME in the initiation and progression of sporadic breast cancer has been well established. But it is suggested that the process of carcinogenesis can be impacted by manipulation of stromal-mediated mechanisms [111]. Histopathological characteristics of *BRCA1* and *BRCA2*-associated tumors are distinct. While *BRCA1*-associated tumors are most commonly a high-grade invasive ductal carcinoma of no special type and the majority fall into the “basal-like” subtype of breast cancer, the *BRCA2*-associated tumors are very similar to sporadically occurring “luminal-type” tumors [112]. *BRCA* signature was previously correlated with a high mutation burden [113], which was specified by distinct mutational landscape in *BRCA1*- and *BRCA2*-deficient tumors revealed by Samstein et al. (discussed below) (Figure 2). Min et al. also showed that *BRCA1* mutation results in higher homologous recombination deficiency scores than *BRCA2* mutation and that germline *BRCA1-* and *BRCA2*-mutated tumors exhibit various differentially expressed genes. They also suggested that differential therapeutic strategies could be effective for *BRCA1*-deficient tumors, which are characterized by upregulation of genes related to EMT, and for *BRCA2*-defficient tumors, where upregulation of genes involved in HER2 signal transduction and in estrogen signaling was observed.

As the *BRCA1* and *BRCA2* gene products are involved in homologous recombination [114], the identification of *BRCA1/2*-deficient tumors in breast cancer patient implied important treatment connotations. The therapeutic strategies, apart from the standard treatment, focused on the increased sensitivity of *BRCA*-deficient cells to DNA damage. For example, platinum salts which act as DNA cross-linking agents were proposed to be more likely associated with chemotherapeutic sensitivity in *BRCA*-deficient tumors. Torissi et al. reviewed the results of the major clinical trials where platinum salts have been used in *BRCA*-associated breast cancer patients in all the settings of disease and confirmed an increased benefit of carboplatin in *BRCA*-associated advanced breast cancer, but not in the neoadjuvant setting in early breast cancer [115]. Another option is use of platinum salts in combination or in sequence with poly(ADP-ribose) polymerase (PARP) inhibitors, which act to limit repair of single strand breaks [116]. A small study of Litton et al. [117] showed that neoadjuvant PARP inhibitor talazoparib, administered as a single-agent in patients with germline *BRCA1/2* mutation, achieved an impressive pathological complete response rate of 50% and supported the larger neoadjuvant clinical trial (NCT03499353). The possible mechanism of action of PARP inhibitors in the TME of *BRCA1*-mutated TNBC was elucidated by Pantelidou et al. The authors demonstrated that the PARP inhibitor olaparib induced CD8+ T cell infiltration and activation through cGAS/STING pathway in tumor cells [118]. Broad analysis of using PARP inhibitors in the treatment of early breast cancer patients with *BRCA1/2* mutation was recently published by Gonçalves et al. [116].

The association between *BRCA1* and *BRCA2* mutation status and survival rates has yielded conflicting results. Haque et al. has shown no difference in the mortality rates comparing *BRCA1* with *BRCA2*-mutation carriers among patients with TNBC [119]. But other studies showed [120,121] that *BRCA1* mutation carrier status was associated with worse prognosis, as well as Samstein et al. who revealed that truncation mutation in *BRCA2*-deficient tumors was associated with clinical benefit.

The impact of *BRCA* mutation status on immune microenvironment in *BRCA1/2*-associated cancers may represent another potential therapeutic target. Mainly because of the fact that the alterations in DNA damage response pathways and mutational load may influence the response to immune checkpoint inhibitors [122]. Moreover, immune cells may represent also a biomarker of therapy response in patients with *BRCA* pathogenic mutations. Grandal et al. [123] already showed a better response to neoadjuvant chemotherapy in *BRCA*-associated luminal subtype with higher lymphocytic infiltration after chemotherapy completion. Comparison of pre-treatment immune infiltration by Sønderstrup et al. [124] showed a greater prevalence of high stromal TILs in *BRCA1*-deficient compared to *BRCA2*-deficient tumors. Features of genomic instability of *BRCA1/2*-deficient cancers including increased mutation burden were showed also by Wen et al. However, they showed that only *BRCA1*-deficient tumors were associated with increased expression of PD-L1 and PD-1 together with higher abundance of tumor-infiltrating immune cells, and enrichment of T cell-inflamed signature [125]. Homologous recombination deficiency (HRD) scores and hormone receptor subtype are predictive of immunogenicity in *BRCA1/2* breast cancers and may aid in designing optimal immune therapeutic strategies, as suggested by Kraya et al. [126].

BRCA1 and BRCA2 modulate the tumor-immune microenvironment also because of distinct mutational landscape, as revealed by Samstein et al. The authors showed on murine model that *BRCA1* and *BRCA2* loss differentially affect the lymphoid compartment of the tumor-immune microenvironment and led to different proportion of various immune system components. The distinct tumor-immune microenvironments in *BRCA1* and *BRCA2*-deficient tumors are modulated by distinct mutational and copy-number profiles, which can predispose to distinct immune checkpoint blockade response. Whole genome sequencing of 4T1 Brca2^null^ cells revealed an accumulation of single-nucleotide variants (SNVs) and insertions or deletions (indels) compared to 4T1 BRCA1^null^ cells [127]. Additionally, others observed that distinct patterns of genomic alterations were associated with *BRCA1-* and *BRCA2*-deficient breast tumors and revealed elevated levels of large rearrangements and tandem duplications in *BRCA1*-deficient tumors, and increased SNVs and indels with microhomology in *BRCA2*-deficient tumors (Figure 2) [128,129]. Samstein et al. also observed specific enrichment of gene expression programs and immune cell populations related to innate immunity in Brca2^null^ tumors relative to BRCA1^null^ tumors. Patient data analysis revealed that truncating germline mutations in *BRCA2*, but not *BRCA1*, were associated with improved overall survival after immune checkpoint blockade. They retrospectively analyzed a cohort of patients profiled with targeted next-generation sequencing (the MSK-IMPACT) and noted a 44.4% rate of clinical benefit among patients receiving immune checkpoint blockade (ICB) with truncating *BRCA2* germline or somatic mutations relative to truncating mutations in *BRCA1* (hazard ratio 0.48, 95% CI 0.29–0.80, *p* = 0.004 for *BRCA2* vs. 0.76, 95% CI 0.48–1.54, *p* = 0.45 in *BRCA1*). [127,130]. These data suggest that the tumor cell-intrinsic differences between *BRCA1* and *BRCA2* deficiency result in differences in immune landscapes, immune cell infiltration of tumors and distinct resistance mechanisms, and require further investigation for the future clinical trials design.

## 6. Conclusions

The tumor microenvironment, as another dimension of tumor complexity, has been acknowledged as an important hallmark of cancer more than decade ago. In many aspects it dictates and impacts the tumor behavior and treatment response. However, how the various cells residing in the TME of germline *BRCA1/BRCA2*-deficient breast tumors that are equally impaired by the same germline mutation contribute to the pathogenesis of the hereditary breast cancer has not been consistently addressed. Results of several experimental and preclinical studies unequivocally point to the need to study not only the tumor epithelial cells, but also the wider concept of TME in which the *BRCA* genes exert their functions. This review aimed to provide significant insights into up-to-date knowledge about the role of *BRCA1/2*-mutated tumor microenvironment and possible mechanisms by which it interacts with and promotes breast cancer development and progression. Uncovering and exposing these mechanisms of the aberrant microenvironment might provide more effective strategies for treatment of germline mutation-carrying breast cancer patients.

## Figures and Tables

**Table 1:** List of proteins with which BRCA1 and BRCA2 interact and their role in DNA repair machinery.

Protein	Binding Partners	Function	Ref.
BRCA1	MRN complex CtIP	DSB end resection	[24]
	RAP80 MERIT40 BRCC36/45 Abraxas	stabilize/maintain DNA damage signaling and promote G2/M checkpoint arrest	[25,26]
	TOBP1 BACH1 (BRIP1/FANCJ)	S-phase cell cycle arrest	[24,27]
	Rad51 FANCD2	repair replication forks stalled at DNA interstrand cross-links	[28]
	MLH1 ATM BLM MRN complex	nucleotide excision repair (NER) pathway	[29]
	PALB2 BRCA2	HR-mediated DNA repair	[30]
BRCA2	BRCA1 RAD51 PALB2	HR-mediated DNA repair	[31,32]

Abbreviations: MRN complex (comprised of MRE11, RAD50 and Nijmegen breakage syndrome protein 1), CtIP (CtBP-interacting protein), DSB (double-strand breaks), TOPBP1 (DNA topoisomerase 2‑binding protein 1), ATM (Ataxia-telangiectasia mutated), PALB2 (Partner and Localizer of BRCA2).
